# The Multiple Faces of MNT and Its Role as a MYC Modulator

**DOI:** 10.3390/cancers13184682

**Published:** 2021-09-18

**Authors:** Judit Liaño-Pons, Marie Arsenian-Henriksson, Javier León

**Affiliations:** 1 Department of Microbiology, Tumor and Cell Biology (MTC), Karolinska Institutet, SE-171 65 Stockholm, Sweden; marie.arsenian.henriksson@ki.se; 2 Departmento de Biología Molecular and Instituto de Biomedicina y Biotecnología de Cantabria (IBBTEC), Universidad de Cantabria-CSIC, 39011 Santander, Spain; leonj@unican.es

**Keywords:** MNT, MYC, MAX, REL, transcriptional regulation, proliferation, cancer

## Abstract

**Simple Summary:**

The MYC oncoprotein is deregulated in up to 70% of human tumors and has a crucial role in the initiation, progression, and maintenance of the tumorigenic process. The MYC modulator MNT belongs to the MXD family of MYC antagonists and transcriptional repressors. It differs from the other MXD family members due to its essential role in the cell and its diverse and complex functions that can either facilitate or impair MYC-driven tumorigenesis. As MYC is a difficult therapeutic target, increasing our knowledge of other proteins in the MYC network will provide the basis for alternative strategies to impair MYC activity in cancer.

**Abstract:**

MNT is a crucial modulator of MYC, controls several cellular functions, and is activated in most human cancers. It is the largest, most divergent, and most ubiquitously expressed protein of the MXD family. MNT was first described as a MYC antagonist and tumor suppressor. Indeed, 10% of human tumors present deletions of one *MNT* allele. However, some reports show that MNT functions in cooperation with MYC by maintaining cell proliferation, promoting tumor cell survival, and supporting MYC-driven tumorigenesis in cellular and animal models. Although MAX was originally considered MNT’s obligate partner, our recent findings demonstrate that MNT also works independently. MNT forms homodimers and interacts with proteins both outside and inside of the proximal MYC network. These complexes are involved in a wide array of cellular processes, from transcriptional repression via SIN3 to the modulation of metabolism through MLX as well as immunity and apoptosis via REL. In this review, we discuss the present knowledge of MNT with a special focus on its interactome, which sheds light on the complex and essential role of MNT in cell biology.

## 1. Introduction

The discovery of MNT (MAX’s Next Tango) was reported by two independent laboratories in 1997. One group found MNT through a two-hybrid screening in yeasts using a murine cDNA library with MAX as the bait [1], while the other group isolated *MNT* (originally called *ROX*) in a search for transcribed sequences of the human chromosome 17p13.3 [2].

The MNT transcription factor belongs to the MXD family (originally called MAD), which shares a basic helix–loop–helix–leucine zipper (bHLHLZ) domain as well as a SIN3 interaction domain (SID). The bHLHLZ is necessary for forming dimers with MAX and for binding to DNA on E-boxes (Enhancer-boxes). The SID establishes the interaction with SIN3 proteins, facilitating transcriptional repression (see below) [3,4]. Notably, the SID repressor domain linked to Cas9 has been described as an efficient tool in CRISPR-mediated repression of targeted genes [5]. The canonical MXD proteins are MXD1, MXI1, MXD3, and MXD4. There is yet another member, MGA [6], that shows little similarity with the other MXDs, as it lacks the SID but binds to MAX through its bHLHLZ domain. MGA is a very large protein (3065 amino acids) and besides the bHLHLZ, it contains a T-box domain, which is described in Brachyury proteins and is a characteristic of the TBX (T-Box) transcription factor family. Its structure allows the regulation of both MAX-network and T-domain target genes, either as an activator or repressor [6,7,8]. Inactivation of *MGA* is frequent in human cancer, and a recent study has demonstrated that MGA functions as a tumor suppressor in murine models of lung carcinoma and in colorectal cancer organoids, partly due to the de-repression of MYC target genes [9].

MNT is a unique member of the MXD family due to several features; it is (i) the largest protein of the family; (ii) the most divergent gene in its sequences; (iii) ubiquitously expressed in human tissues; (iv) essential for mouse development; (v) expressed in both quiescent and proliferating cells; (vi) altered in human cancer; and (vii) is frequently involved in tumorigenesis (Table 1). It also plays an important role in modeling the oncogenic activities of MYC, as an antagonist in some models or as a cooperator in others (reviewed in [10]).

MNT is evolutionarily conserved, an argument for its relevant role in cell biology. It is the sole MXD protein identified in the fly *Drosophila melanogaster* [23]. Flies express a “minimal” network composed of dMax, a single MYC protein (dMyc), and a sole MXD protein (dMnt). In general, MNT and MXD1 are present along the phylogenetic tree, whereas the other MXD are only present in vertebrates (reviewed in [15]). Interestingly, dMnt is recruited to a higher number of *Drosphila* genes than dMyc is [24]. MNT orthologues are found in primitive pluricellular organisms from the Cnidaria (*Hydra* spp., *Nemastotella* spp.) and Placozoa (*Trichoplax* spp.) phylum [25]. In contrast, the nematodes *C. elegans*, *C. briggsae*, and *Brugia malayi* lack MNT or MYC orthologs. However, these species, together with the trematode *Schistosoma mansoni*, present orthologs for MAX, MLX, MXD, and MLXIP [15,25,26].

In contrast to the other members of the MXD family, MNT is an essential protein during development, as *Mnt* knockout mice die soon after birth [11,27]. Mice with null homozygous deletions for *Mxd1*, *Mxd2,* or *Mxd3* show different defects [12,13,28] but survive to adult ages. *Mnt*-deficient embryos exhibit small size throughout development and, interestingly, show reduced levels of c-Myc and N-Myc [11]. *Mnt*-overexpressing embryos also have developmental defects, are of a smaller size, and have decreased cellularity [1]. These data suggest that a tight control of MNT levels is necessary for the correct development.

## 2. MNT Structure and Regulation

**Figure 1:**
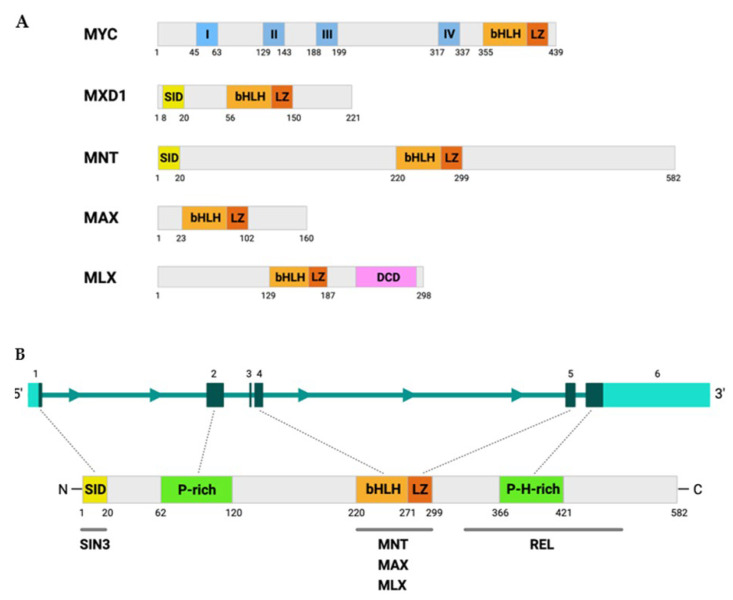
MNT and MYC protein structure. (**A**) Comparison between MYC, MXD1, MNT, MAX, and MLX protein sizes and domains. The canonical isoforms are shown; specifically, the p22 isoform for MAX and the gamma isoform for MLX. MYC boxes are represented in blue with the numbers I–IV. (**B**) The *MNT* gene structure is schematized at the top, with boxes representing the six exons, and a scheme of the MNT protein at the bottom. The *MNT* exons that encode the most important regions are indicated as boxes, and the dotted lines connect the exons with the corresponding protein domains. The main MNT-interacting proteins are shown at the bottom. SID: SIN3 interacting domain; P-rich: proline-rich sequence; bHLH: basic helix–loop–helix; LZ: leucine zipper; P-H-rich: proline and histidine-rich region; DCD: dimerization and cytoplasmic localization domain. Figures were created with BioRender.com (Toronto, ON, Canada).

The *MNT* gene is localized at human chromosome 17p13.3 and mouse chromosome 11B5. The human gene is 1.7 kb long and contains six exons according to the NCBI Gene browser (GRCh38/hg38). Human *MNT* encodes a 582-amino acid protein that contains (i) a coiled-coil α-helix structure for interaction with SIN3 (SID); (ii) a highly proline-rich region, similar to the activation domains of other transcription factors like MYC; (iii) a bHLHLZ domain, for homodimerization, interaction with MAX and MLX, and DNA-binding at E-boxes, and (iv) a proline and histidine-rich region at its C-terminal domain [1,2]. Proline-rich regions are usually involved in protein–protein interactions [29] and are typical of transcriptional activation domains and are absent in the other MXD proteins. The SID is necessary for the repressive function of MNT, as its deletion turns the protein into a transcriptional activator [1]. We recently described that the C-terminal domain of MNT (amino acids 306–537) is necessary for its interaction with REL [30], a member of the NF-κB family. The MNT, MYC, MXD1, MAX, and MLX proteins are schematized in Figure 1A. The *MNT* gene, protein, and its main interactors are shown in Figure 1B.

The basic region of MNT differs from the other bHLHLZ proteins in the proximal MYC network, and MNT–MAX dimers show a higher preference for non-canonical CACGCG E-boxes though they can also bind to the canonical CACGTG [2]. This difference in the basic regions of the DNA explains why MNT has a subset of unique target genes apart from the ones shared with MYC and the other MXDs.

MNT is a mainly nuclear protein with a half-life of 30–60 min, and it appears as a doublet of 72–74 kDa in Western blots [1,2]. The 72 kDa form is the form detected in growth-arrested cells. In contrast, MNT is detected at 74 kDa in serum-stimulated cells and corresponds to the hyperphosphorylated protein. This phosphorylation is conducted by MKK/ERK kinases and impairs the interaction with SIN3B and, consequently, the repressive function of MNT [31]. So far, no other post-translational modifications have been described for MNT except for ubiquitination (described below) [30].

## 3. MNT Expression

**Figure 2:**
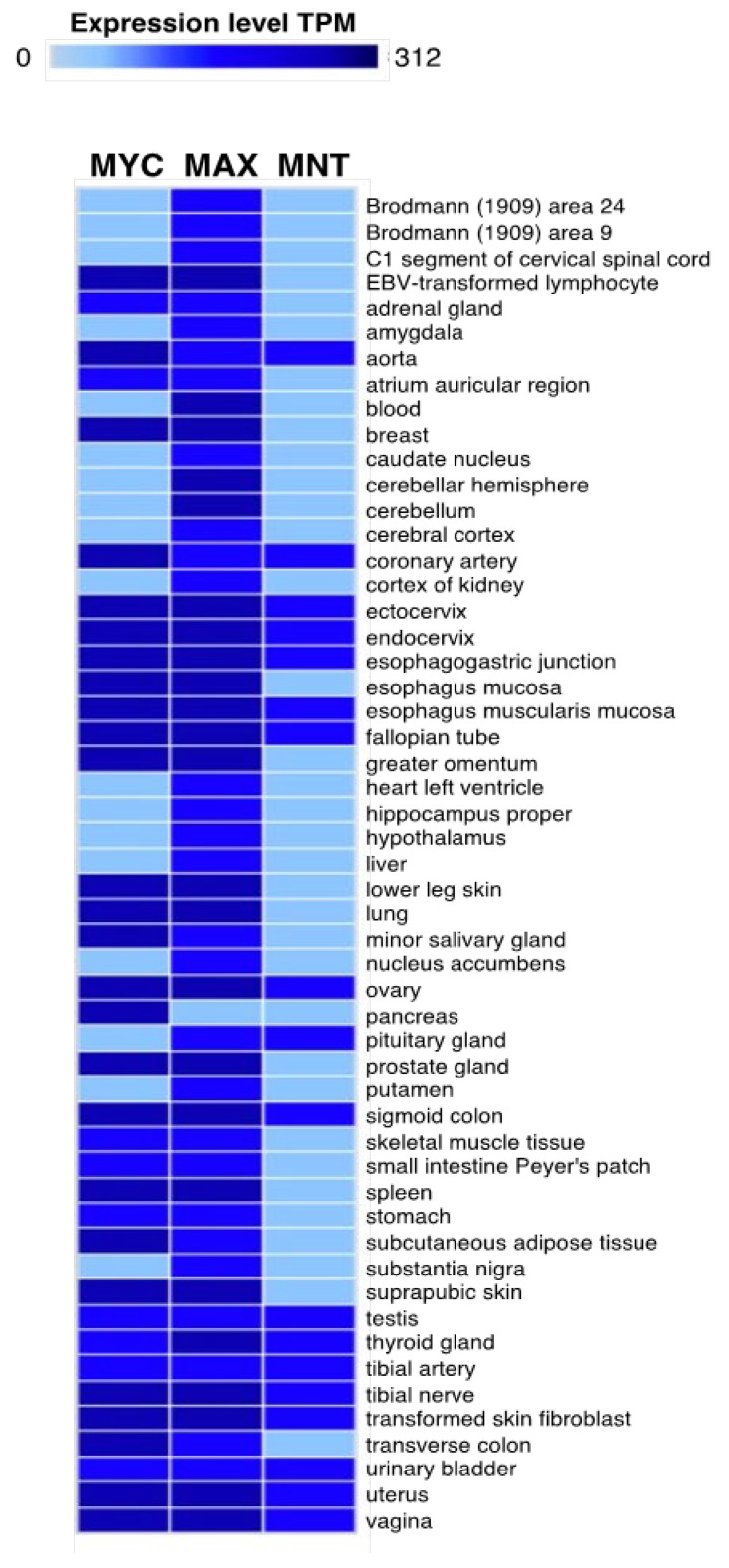
Expression of *MYC*, *MAX*, and *MNT* in human tissues. Data obtained using the GTEX Portal (gtexportal.org). TPM, transcripts per million.

*MNT* mRNA is expressed in most human tissues according to a massive RNA-sequencing study [32]. *MNT* mRNA levels were highest in the bone marrow, brain, endometrium, spleen, and testis, while the lowest levels were in the heart, liver, pancreas, and salivary glands. The GTEX database gives a more restricted pattern of *MNT* mRNA expression in human tissues, peaking in the esophagus, intestine, and pancreas (Figure 2).

*Mnt* is broadly expressed during mouse development. Its mRNA has already been detected in early conceptus and in multiple tissues during development (informatics.jax.org/marker/MGI:109150) until stage E10.5. At later stages, *Mnt* expression is the highest in neural structures [1].

During *Xenopus laevis* development, the MNT orthologue *XMnt* is expressed in early embryos at the neurula stage and in migrating cranial neural crest cells [33]. The expression of Mnt in the neural tissues of mice and *Xenopus* seems consistent with the cleft palates and craniofacial deformities observed in *Mnt*-deficient mice [11,34]. In *Xenopus*, *XMnt*, *XMad1,* and *XMad3* exhibit distinct areas of expression during embryogenesis, suggesting non-redundant functions [33].

Both quiescent cells and fibroblasts express *MNT* throughout the cell cycle. MNT levels do not increase upon serum stimulation of quiescent cells [31]. This is in contrast with the expression pattern of *MYC* (expressed mainly in proliferating cells) and most other MXD proteins, such as *MXD1*, *MXI1*, and *MXD4* (mainly expressed in quiescent cells). Thus, MNT–MAX dimers coexist with MYC–MAX in proliferating cells [1]. MNT represses its own expression in a MAX-dependent manner by direct binding to its promoter. E-box 2, which is mapped at −788 bp from the transcription start site, is critical for this autoregulation. A decrease or absence of MAX results in high levels of MNT, which are distributed among cytoplasm and nucleus. MNT also binds weakly to its own promoter in the absence of MAX [35]. The presence of E-boxes in the *MNT* promoter indicates that other bHLHLZ proteins could also control its expression.

MNT protein levels are also regulated by the E6-associated protein (E6AP), an E3 ubiquitin ligase that induces its ubiquitination and degradation by the proteasome. This was observed in myeloid differentiation experiments, in which all-trans retinoic acid, vitamin D3, or phorbol 12-myristate 13-acetate downregulated E6AP and consequently reduced the E6AP-mediated degradation of MNT [30].

## 4. MNT Interactions in the Proximal MYC Network

**Figure 3:**
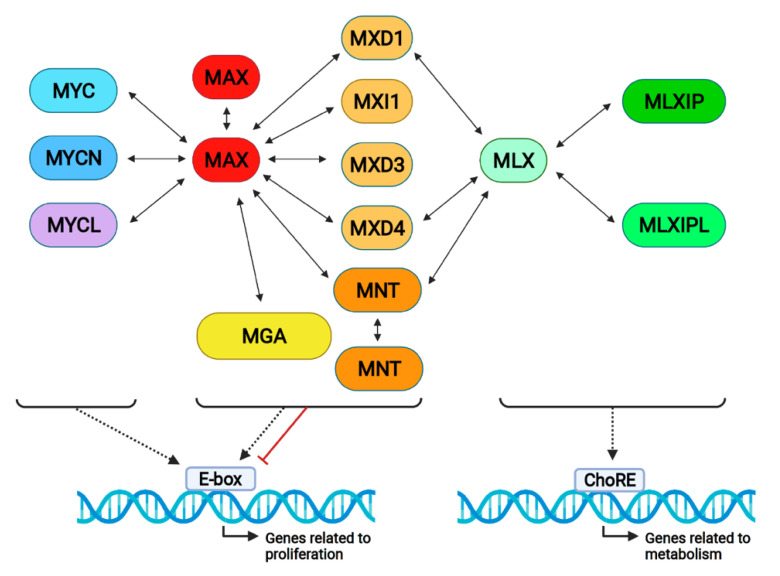
The proximal MYC network and its interactions. MYC proteins form dimers with MAX and bind DNA on E-boxes, generally activating transcription. All MXD proteins bind to MAX and MXD1, and MXD4 and MNT also bind to MLX, activating or repressing gene transcription. MLX forms complexes with MLXIP (MONDOA) and MLXIPL (MONDOB), binding on ChoRE (carbohydrate response elements) and regulating metabolic genes. Notably, MAX and MNT form homodimers, as represented in the Figure.

The proximal MYC network is composed of thirteen proteins distributed into three protein families: MYC, MXD, and MLXIP. The MYC family includes c-MYC (also called MYC), MYCN (or N-MYC), and MYCL (or L-MYC). MAX connects MYC with the MXD proteins while MLX connects the MXD with the MLXIP (also known as MONDO) proteins. A scheme of this network is shown in Figure 3.

In contrast to the MYC and the MXD proteins, MNT can form homodimers. This was initially discovered in yeast two-hybrid assays and with recombinant proteins [1,2]. More recently, the formation of MNT dimers has been shown in rat and human cell lines. Yet, MNT–MAX complexes are formed preferentially to MNT–MNT dimers [35]. Electrophoretic mobility shift assay (EMSA) assays with in vitro synthesized proteins also showed that MNT–MNT can bind to E-boxes but with a lower affinity compared to MNT–MAX [2]. Importantly, it has been shown that MNT homodimers not only bind DNA, but they are also able to regulate gene transcription [35].

In summary, MNT can form heterodimers with MAX [1,2], heterodimers with MLX [36], or homodimers [35] through the bHLHLZ domain (Figure 3). Thus, MNT serves as a bridge between MYC–MAX (the proliferative arm of the network) and MLX–MLXIP/MLXIPL (the metabolic arm of the network) [37].

## 5. MNT Interactions Outside the Proximal MYC Network

### 5.1. SIN3 Proteins

SIN3A and SIN3B are transcriptional corepressors that recruit histone deacetylases and other chromatin modifying enzymes, inhibiting transcription upon binding to E-boxes. MNT represses transcription through direct interaction with the SIN3 proteins through the SID, a domain in the N-termini that binds to the SIN3 s paired amphipathic helix (PAH2) domain of SIN3B [3,22]. SIN3A and SIN3B, in turn, recruit histone deacetylases through their histone interaction domain (HID) or co-factors including N-CoR, SDS3, SAP30, SAP18, RBP1, or ING1/2 that work by bridging and stabilizing the complex and/or by enhancing the chromatin remodeling activities [4,38]. In the case of MXD1, this is mediated by the recruitment of HDAC1/3 by SIN3 [39]. The ability of MNT to block MYC-dependent transformation and cell cycle progression is lost in SID mutants, indicating a close link between biological function and repression [1,40].

### 5.2. REL

Recently, we identified the NF-κB family protein REL (also known as c-REL) as a MNT interacting partner through the proteomic analysis of MNT co-immunoprecipitating proteins in a MAX-independent setting. MNT acts as a repressor of the NF-κB pathway by two suggested mechanisms: (i) the retention of REL in the cytoplasm in REL–MNT complexes and (ii) the repression of REL target genes [41].

Once a stimulus is detected by the cell, the NF-κB pathway is activated and IκBα is degraded. However, NF-κB also induces a negative feedback loop, leading to the transcription of *NFKBIA*/*IκBα*. The newly synthesized IκBα enters the nucleus and shuttles the NF-κB dimers back to the cytoplasm to terminate transcription [42,43,44]. MNT–REL can bind and repress the *NFKBIA/IκBα* gene at least in some cell types. Therefore, MNT might be acting to limit NF-κB activity in the absence of specific pathway activators. In addition, the requirement of MNT for cell proliferation is dependent on REL in two cellular models. This indicates that the pro-survival role of MNT can be mediated through REL. Interestingly, MNT regulates the NF-κB pathway independently of MAX [41].

### 5.3. MATα-1

Methionine adenosyltransferase a1 (MATα-1) forms a complex with MNT–MAX in the normal liver and bile duct epithelial cells that binds E-boxes. In cholestasis and cholangiocarcinoma cells, MATα-1-MNT interaction drops, and there is a switch towards MYC and MAF protein complexes [45]. The switch from MNT–MAX to MYC–MAX during cholestasis has been previously described, and it is responsible for p53 and cyclin D1 up-regulation and apoptosis [46].

### 5.4. Other Proteins

Recent studies have identified at least four other proteins as MNT interactors [41]: CCDC6, AMPD2, QSER1, and TPP2. CCDC6 is involved in DNA damage response [47]; AMPD2 participates in purine metabolism as an AMP deaminase [48]; QSER1 has recently been described as a demethylation regulator [49]; and TPP2 is a peptidase with roles in antigen processing, lipid metabolism and CCK8 hormone regulation [50].

**Figure 4:**
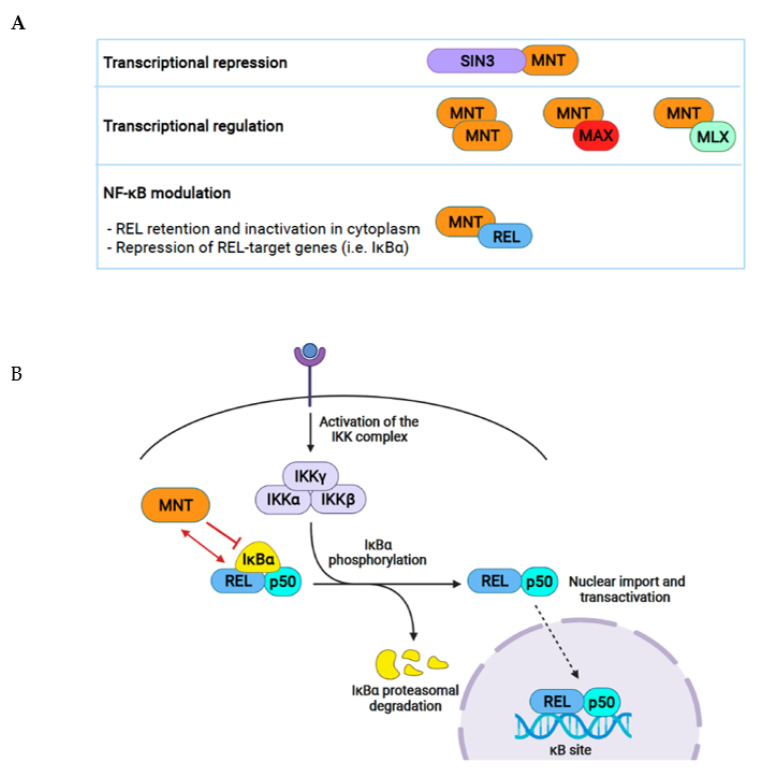
MNT functional interactions. (**A**) MNT represses transcription through SIN3, which recruits a HDAC complex and turns DNA into a closed conformation. MNT homodimers and MNT–MAX and MNT–MLX heterodimers regulate transcription by binding DNA at E-boxes. MNT–REL interaction results in NF-κB regulation by retaining REL dimers inactive in the cytoplasm and by repressing REL target genes that would usually be activated by REL. (**B**) Once the cell receives an NF-κB activation signal, the IKK complex is activated and is able to phosphorylate IκBα, which is bound to REL-p50 dimers in the cytoplasm. This phosphorylation targets IκBα for ubiquitination and posterior proteasomal degradation. Finally, the REL-p50 dimers are free to translocate into the nucleus and regulate their target genes by binding to κB sites on the DNA. MNT forms complexes with REL and inhibits its functions.

The main MNT functional interactions are represented in Figure 4A and include NF-κB modulation by MNT in Figure 4B.

## 6. MNT Alterations in Cancer

*MNT* has been classified both as an oncogene or as a tumor suppressor gene based on its diverse activities in the cells. The data arguing that it behaves as tumor suppressor gene can be summarized as follows: (a) the tissue-specific loss of *Mnt* in mouse models results in thymic lymphoma [51] or mammary tumors [27,52] (discussed below in point 7.1); (b) MEFs deficient in *Mnt* proliferate faster than wild-type cells and prematurely enter into the S phase [27] (discussed below in point 7.1); (c) in a pan-cancer study of The Cancer Genome Atlas (TCGA) data, heterozygous *MNT* deletion was found in 10% of the tumors (overall frequency) and in more than 20% of liver hepatocellular carcinomas, lung adenocarcinomas, sarcomas, and uterine carcinosarcomas [16].

The *MNT* locus at chromosome 17p13.3 is a hot spot for the loss of heterozygosity (LOH) in tumors such as sporadic breast cancer, medulloblastomas, or chronic lymphocytic leukemia (CLL) [21,53,54,55] as well as in ovarian cancer [56], astrocytomas [57], bladder cancer [58], or osteosarcoma [59]. However, no inactivating mutations in *MNT* have been found in these tumors. *MNT* deletions were also found in acute lymphoblastic leukemia [20], and a decrease in its levels in medulloblastomas, which was caused by the haploinsufficiency of chromosome 17p13.3 [21]. In the Sézary Syndrome, a malignant variant of cutaneous T-cell lymphoma, more than 60% of the patients show a loss of one or both *MNT* alleles [17,18], thus being the tumor with the highest frequency of *MNT* deletions. In agreement, the down-regulation of *MNT* expression has also been reported in Sézary Syndrome [19].

The analysis of the genomic data available in TCGA shows that 0.6% of tumors carry *MNT* mutations, with missense mutations being the most common. Most of these missense mutations are observed in colorectal, endometrial, melanoma, esophagic, and head and neck cancer. The majority of mutations map in the C-terminal half of the protein, i.e., downstream of the bHLHLZ domain.

**Figure 5:**
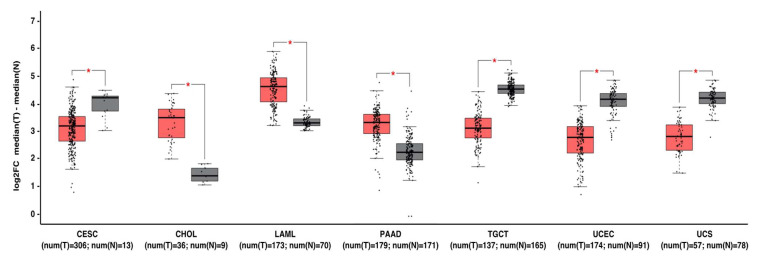
MNT can act as a tumor suppressor gene or oncogene depending on the tumor type. Gene expression profiling interactive analysis (GEPIA, http://gepia.cancer-pku.cn/, accessed on 7 July 2021) was performed to validate *MNT* mRNA expression in selected cancer type samples (in red) vs. normal samples (in grey). The expression data were first log2(TPM+1) transformed for differential analysis, and the log2FC was defined as median (tumor)–median (normal). Data are represented as mean ± SD (* *p* < 0.01). Abbreviations: CESC, cervical squamous cell carcinoma and endocervical adenocarcinoma; CHOL, Cholangiocarcinoma; LAML, acute myeloid leukemia; PAAD, pancreatic adenocarcinoma; TGCT, testicular germ cell tumors; UCEC, uterine corpus endometrial carcinoma; UCS, uterine carcinosarcoma.

Analysis of *MNT* mRNA expression in tumors shows that it is down-regulated in tumor samples compared to their normal tissue counterparts, including testicular germ cell tumors, uterine corpus endometrial carcinoma, and uterine carcinosarcoma. However, the opposite occurs in other tumor types, including cervical carcinoma, cholangiocarcinoma, acute myeloid leukemia, and pancreatic adenocarcinoma (Figure 5). These differences likely reflect the complex biological activities of MNT, which depend on the cellular context and the repertoire of proteins to interact with.

## 7. Functions of MNT in the Cell

**Figure 6:**
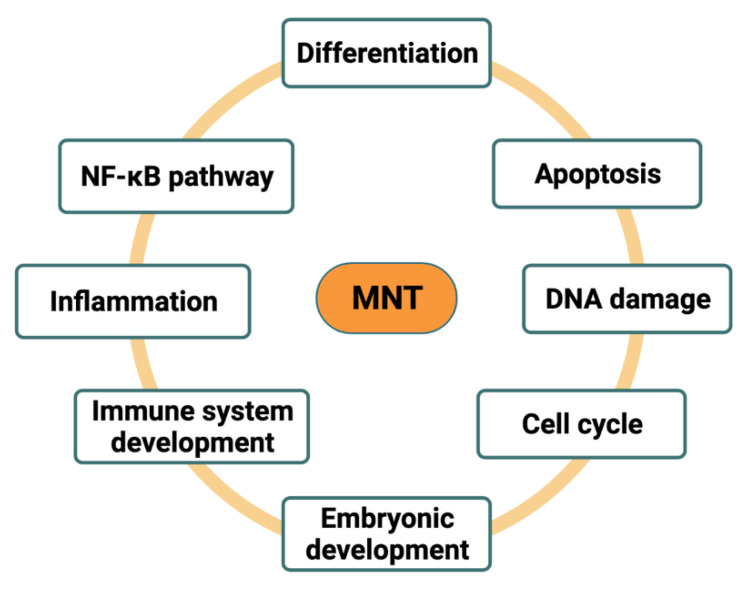
Cellular functions of MNT. MNT is involved in several cellular functions, including differentiation, apoptosis, DNA damage, cell cycle, embryonic development, immune system development, inflammation, and NF-κB pathway regulation. Depending on the model, MNT will either antagonize or cooperate with MYC.

MNT plays a pivotal role in controlling cell proliferation, differentiation, and cellular transformation. The cellular processes that MNT regulates are represented in Figure 6 and are described below. Its functions can result in either antagonism or cooperation with MYC.

### 7.1. MNT as MYC Antagonist

The expression of MNT is ubiquitous, and its levels are constant throughout the cell cycle. Thus, MNT–MAX dimers coexist with MYC–MAX dimers along all phases of the cell. The antagonism between MNT and MYC is achieved at three different levels: (i) competition for forming complexes with MAX, as MNT and MYC show similar affinities towards MAX; (ii) binding to the E-Boxes of their shared target genes; and (iii) the transcriptional repression of genes that are normally activated by MYC–MAX [1,24]. In fact, it has been hypothesized that a major role of MYC is to overcome MNT transcriptional repression rather than its transactivation capacity (reviewed in [22]).

MNT–MYC counteraction was initially studied in wild-type mouse embryonic fibroblasts (wt MEFs) versus *Mnt* knockout fibroblasts (*Mnt*^-/-^ MEFs). *Mnt-*deficient MEFs proliferated faster and entered the S phase prematurely, which was accompanied by an increase in Cdk4 and Ccne1 (Cyclin E) and a decrease in Myc. *Mnt*^-/-^ MEFs have higher apoptotic rates and can efficiently escape senescence compared to wt MEFs. Notably, *Mnt*^-/-^ MEFs can be transformed by oncogenic RAS alone, mimicking cells with *Myc* overexpression. The deletion of *Myc* in MEFs causes a proliferation arrest that can be partially rescued by the simultaneous deletion of *Mnt* [27,60,61,62].

In serum-stimulated quiescent cells, Myc is induced at the G0 to G1 transition, whereas Mnt levels remain constant. During this transition, there is a switch from Mnt–Max to Myc–Max dimers, which activate cell cycle progression genes, such as *Cdk4*, *Ccnd2* (Cyclin D2), *ODC*, or *E2F2*. Both *Mnt* overexpression or *Myc* loss blocks cell cycle entry, suggesting that the ratio of Mnt versus Myc levels could determine the quiescent or proliferative state of the cell [62].

Additional data supporting a MYC–MNT antagonism derive from studies using mice with the conditional deletion of *Mnt* in mammary tissue. This revealed an important role of MNT in mammary epithelium development and tumorigenesis. These mice carry a floxed *Mnt* gene and a *Cre* transgene under the control of the mouse mammary tumor virus (MMTV), which drives *Cre* expression in mammary epithelium. The conditional deletion of *Mnt* in this model led to the formation of an adenocarcinoma with a tumor latency of 6–20 months, similar to the results of *My*c overexpression in mammary epithelium. Indeed, the mRNA expression patterns of mammary tumors resulting from *Mnt* deletion or *Myc* overexpression were very similar [27,52].

The conditional deletion of *MNT* in T-cells caused an increase in both proliferation and apoptosis. This was accompanied by tumor formation with a long latency, the disruption of T-cell development, and the enlargement of the secondary lymphoid organs. There was a modest increase in the expression of Cdk4 and the cyclins D2, E1, A, and B1 and a slight downregulation of Bcl-2 and Bcl-xL. The polarized differentiation of CD4^+^ T cells into Th_1_ (T-helper cell type 1) caused inflammation and, consequently, predisposition to T-cell lymphoma [51,63].

Studies in *Drosophila melanogaster* also show similar MYC–MNT antagonism to the one found in mouse models and in humans. Indeed, dMnt and dMyc have opposing activities in cell growth in vivo [23,24]. There is an important overlap between the dMyc, dMnt, and dMax DNA binding regions, revealing shared target genes. For instance, dMnt antagonized the growth stimulatory effects of dMyc by downregulating pre-rRNA synthesis [64,65]. In this model, dMnt overexpression rescued the viability and cell growth defects caused by *dMyc* deletion.

However, both in mice [52] and in *Drosophila* [24], the overlap between Myc- and Mnt-regulated genes is only partial. This has been attributed to at least two facts: (i) the sequence of the bHLH domains and thus the affinities for DNA are not identical, and (ii) whereas Myc can only bind to Max, Mnt may form at least three different complexes, as discussed in Section 4.

Another proof of MNT and MYC antagonism is the response to hypoxia, a common feature in solid tumors. During hypoxia there is an increase in HIF-1α and HIF-2α, which, in turn, induce the expression of the microRNA *miR-210*. Since *miR-210* downregulates MNT, its levels are lower in hypoxia. Furthermore, MNT downregulation has caused a switch to MYC–MAX dimers and the activation of MYC-target genes in several models. Consequently, tumoral cells can override cell cycle arrest and apoptosis by releasing MNT–MYC antagonism [46,66,67,68]. Hypoxia has an important impact on the cells, which is in part because of the impairment of the circadian rhythm [69]. Apart from the roles of HIF-1α and mTOR in the regulation of the transcriptional clock program, the disturbed MYC/MAX/MNT balance can lead to changes in the expression of circadian rhythm genes. In fact, MNT–MAX were recently described to bind to the *PER2*, *CRY1*, and *CRY2* promoters [70].

MNT–MYC counteraction was also shown in a myeloid differentiation model described by Kapoor and colleagues [30]. Treating HL60 cells with differentiating agents, including all-trans retinoic acid, led to a decrease in E6AP and subsequently to a loss of E6AP-mediated degradation of MNT. In this model, increased levels of MNT antagonized MYC and induced cell cycle arrest followed by myeloid differentiation.

Niu et al. [71] described that MYC induced *miR-378a-3p*, which, in turn, down-regulated MNT in Burkitt’s lymphoma. MNT reduction releases MNT–MYC antagonism and enables the MYC-driven transformation process.

Altogether, these data support the role of MNT as a MYC antagonist and show how MNT restricts the pro-proliferative activities of MYC.

### 7.2. MNT as MYC Cooperator

In contrast to the data presented above, *MNT* can also act as an oncogene cooperating with *Myc* as: (a) T-cell-specific homozygous *Mnt* deletion prevented thymic lymphoma development in mice overexpressing the Myc protein in T cells; (b) *Mnt* heterozygosity slowed Myc-driven tumorigenesis; (c) *Mnt* deletion in homozygosis impairs lymphomagenesis in *Eμ-Myc* mice.

The pro-tumorigenic role of MNT is supported by its pro-survival functions, which seem necessary for sustaining MYC activities. In the study of Link et al., Mnt was necessary for Myc-driven thymic lymphomagenesis in mouse models [72]. *Mnt* knockout together with *Myc* overexpression in the thymocytes resulted in higher apoptotic levels than in cells with *Mnt* knockout or with *Myc* overexpression alone. This was not related to increased p53 but rather to enhanced levels of reactive oxygen species (ROS). Mnt deficient thymocytes were highly sensitive to the inhibition of several ROS detoxification systems. This effect on survival became worse when MYC levels increased, as shown by using models from no MYC to endogenous levels and further to ectopic high MYC expression [73], which is in line with the fact that that MYC-driven proliferation and apoptosis are triggered by certain MYC thresholds. Consequently, MYC levels influence MYC outputs; thus, they determine the differential need for MNT [73].

The pro-survival functions of MNT and its counteracting role on MYC were also described in the response of T-cells to OX40, a T-cell costimulatory molecule from the tumor-necrosis receptor ligand family [74]. This ligand produces an increase in Myc but also in Mxd4 and Mnt. Myc drives T-cell proliferation while Mxd4 and Mnt evade cell death inflicted by Myc excess.

Another study showed that *Mnt* heterozygosity slows down *Myc*-driven B and T-lymphomagenesis in two *Myc* transgenic models, *vavP-MYC10* mice, and *Eμ-myc* mice [75]. However, the exact mechanism was not defined, as the authors did not find changes in the preleukaemic populations, cell size, cell cycle, apoptosis, or senescence of the murine lymphoma cells. In a second study [76], *Mnt* was deleted in homozygosis using two *Mnt* conditional knockdown models: the *Mnt^fl/fl^*;*Eμ-Myc*/*Rag1Cre* and *Mnt^fl/fl^*;*Eμ-Myc/CreERT2* mice, to avoid the early embryonic lethality of homozygous *Mnt* deletion. In the *Mnt^fl/fl^;Eμ-Myc/Rag1Cre* model, the mice carried floxed *Mnt* alleles and the *Rag1Cre35* transgene, which allowed the expression of the Cre recombinase in lymphoid progenitor cells only. In the *Mnt^fl/fl^;Eμ-Myc/CreERT2* model, the *CreERT2* transgene encoded a Cre recombinase fused to the modified hormone-binding domain of the estrogen receptor in the *Rosa26* locus. Thus, Cre recombinase expression can be induced by 4-hydroxy(4OH)-tamoxifen. Both the *Mnt^fl/fl^;Eμ-Myc/Rag1Cre* and *Mnt^fl/fl^;Eμ-Myc/CreERT2* models showed that *Mnt* loss impairs the characteristic Myc-driven lymphomagenesis of *Eμ-Myc* mice, mainly affecting the premalignant early B lineage cell proliferation. *Mnt* deletion also impaired normal lymphopoiesis in wild-type mice, suggesting that MNT has an important role in B-cell development. MNT anti-apoptotic functions must be key to compensate for increases in MYC levels during lymphopoiesis. In MYC-driven B lymphomagenesis, the IL-7 receptor signaling up-regulates both Mnt and Myc, and Mnt suppresses apoptosis by repressing *Bim* thus facilitating MYC activity.

## 8. MAX-Independent Roles of MNT

MAX is an essential protein that supports the functions of the proximal MYC network, as it is a shared partner between the MYC and MXD proteins. Little is known about the function of these proteins in the absence of MAX. However, MAX is deleted in some cancers of neuroendocrine origin, such as pheochromocytomas, paragangliomas, gastrointestinal stromal tumors, and small cell lung cancer [77,78,79,80,81]. This raises the question of how MYC and MXD proteins can conduct their functions without MAX.

We studied the MAX-independent functions of MNT using UR61 cells, which derive from the rat pheochromocytoma PC12 cell line. PC12 cells express a truncated Max protein that lacks helix 2, the leucine zipper, and the C-terminal region. Thus, Max in PC12 cannot homo- or heterodimerize, nor repress transcription [82]. In this model, we observed that *Mnt* expression is higher in the absence of Max and that it distributes in both the nucleus and the cytoplasm, in contrast to *MAX*-expressing cells, in which MNT is mostly nuclear [1,2]. This is due to the negative regulation of Mnt–Max dimers over the *Mnt* promoter. Thus, in the absence of Max, Mnt cannot autoregulate its expression and the excess of Mnt spreads towards the cytoplasm. *Max* re-expression led to a decrease of Mnt levels, as Mnt–Max dimers repress the *Mnt* promoter. Interestingly, Mnt was required for sustaining cell proliferation even in the absence of Max and regulated gene expression, especially DNA damage and cell cycle genes, in a Max-independent way. We confirmed Mnt–Mlx heterodimerization and Mnt homodimerization in UR61 cells. The latter had only been shown in yeast two-hybrid assays and in vitro using recombinant proteins [1,2]. Chromatin immunoprecipitation (ChIP)-qPCR and ChIP-seq revealed that Mnt binds to the DNA on E-boxes in the absence of Max. We also observed Mnt binding to fork head factors sites, in accordance with a study on the coordinated regulation of MNT and FOXO of some cell cycle control genes [83]. Some of the Mnt direct target genes that we detected were *BIRC5*/Survivin, *CDK1*, *BRCA1*, *ERCC6*, and *FBXO32*, the latter of which was previously described [83]. Despite this, we believe that in the absence of MAX, MNT binding to the DNA is weaker, as the overall results of our ChIP-seq in Max-deficient cells was low compared to other available ChIP-seq data for MNT in *MAX*-expressing cells (ENCODE project) [35]. This is in line with results in wild-type and knockout *Max* B-cells, where the genomic occupancy of Mnt decreases in the absence of Max, although it does not disappear, as is the case for Myc [84].

As described above, MNT can form dimers with MLX, which, in turn, interact with MLXIP and MLXIPL. These complexes play an important role in the regulation of the genes involved in glycolytic and lipogenic metabolism by binding to ChoRE elements, which are composed of two E-boxes separated by five nucleotides (Figure 3) [85]. Thus, it is conceivable that MNT impinges on metabolism by modifying the levels of MLX available for binding to MLXIP or MLXIPL.

## 9. Concluding Remarks and Therapeutical Perspectives

MYC has been one main focus of cancer research since it was identified in 1977–1979 as the oncogene captured by an avian oncogenic retrovirus [86,87,88] and was later found deregulated in 70% of human tumors [89]. However, in recent years, the interest in understanding the entire proximal MYC network has grown. The studies mentioned in this review place MNT in a key position as a MYC modulator and demonstrate its diverse and essential roles in cell biology (summarized in Figure 6).

The functions of MNT are generally connected to MYC. Yet, studies in *MYC* knockout cells raised the question of whether MYC is a MNT antagonist rather than MNT a MYC antagonist [22]. *Mnt* knockdown in *Myc*-deficient rat fibroblasts showed that Mnt also conducts pro-survival functions in the absence of Myc [61]. Thus, the most important role of MYC might be to relieve MNT-mediated repression rather than activating transcription. In this scenario, MNT functions would remain unaffected by the presence or absence of MYC, while excessive MYC levels would impact MNT by impairing its transcriptional repressing activity.

The dual role of MNT in cancer, either as a MYC antagonist and or as a MYC cooperator, offers the possibility of using MNT as a therapeutical target. For instance, in MYC-driven lymphomas, tumor cells depend on MNT to survive. Thus, drugs that inhibit MNT activities (*e.g.*, impairing critical interactions) would increase the efficacy of current therapies by enhancing apoptosis [76]. One possibility would be to test the MYC:MAX inhibitors Omomyc, 10058-F4, 10074-G5, Mad, or MYCMI-6, 11, and 14 on MNT. Some of these, such as Omomyc or Mad peptides, preferentially bind to MAX and not to MYC. Hence, they might be used as MNT inhibitors by impairing MNT–MAX interaction. However, this possibility has not been formally addressed yet [89,90,91,92,93]. In addition, as MNT downregulation stimulates NF-κB signaling through the interaction with REL (Figure 4B), NF-κB inhibitors could be used in tumors with MNT loss, such as in CTCL (Table 1). Further research will uncover new roles of MNT in cell biology and will provide strategies for its use as a clinical target.

## Figures and Tables

**Table 1:** Main differences between the human MNT, other human MXD proteins, MGA, and MYC. The sizes of the proteins are the largest canonical forms, according to the UniProt database. Aa, amino acids; ND, not done; DEL, deletions; AMP, amplifications; MUT, mutations; CTCL, Cutaneous T-cell lymphoma; MB, medulloblastoma; SS, Sezary Syndrome; ALL, acute lymphoblastic leukemia.

	MNT	MXD1	MXI1 (MXD2)	MXD3	MXD4	MYC	References
**Protein size**	582 aa	221 aa	228 aa	206 aa	209 aa	439 aa	Uniprot database
**P-rich sequences**	Yes	No	No	No	No	Yes	[2]
**Phenotype of KO mice**	Perinatally lethal/Craniofacial abnormalities	Viable/Increased immature granulocyte progenitors	Viable/Hyperplasia	Viable/Enhanced sensitivity to apoptotic stimuli	ND	Embryonic lethal E9.5–E10.5	[11,12,13,14]
**Proximal MYC Network interactors**	MNT, MAX, MLX	MAX, MLX	MAX	MAX	MAX, MLX	MAX	Reviewed in [15]
**Expression in cells**	Quiescent and proliferating	Quiescent	Quiescent and proliferating *	Proliferating (S-phase)	Quiescent	Proliferating	Reviewed in [15]
**Pan-cancer copy number alterations (%)**	DEL 10 AMP 3 MUT 1	DEL 2 AMP 6 MUT < 0.5	DEL 8 AMP 4 MUT < 0.5	DEL 7 AMP 8 MUT < 0.5	DEL 6 AMP 5 MUT < 0.5	DEL 2 AMP 21 MUT 1	[16]
**Involvement in human cancer**	DEL in CTCL-SS and ALL. Reduced expression in MB	None or weak	None or weak	None or weak	None or weak	Strongly, 70% of tumors have deregulation	[17,18,19,20,21,22]

* central nervous system, epidermis, and the myeloid lineage.
